# New paradigms on hematopoietic stem cell differentiation

**DOI:** 10.1007/s13238-019-0633-0

**Published:** 2019-06-14

**Authors:** Hui Cheng, Zhaofeng Zheng, Tao Cheng

**Affiliations:** 1 State Key Laboratory of Experimental Hematology, Chinese Academy of Medical Sciences and Peking Union Medical College, Tianjin, 300020 China; 2 Institute of Hematology and Blood Disease Hospital, Chinese Academy of Medical Sciences and Peking Union Medical College, Tianjin, China; 3 Center for Stem Cell Medicine, Chinese Academy of Medical Sciences, Tianjin, China; 4 Department of Stem Cell and Regenerative Medicine, Peking Union Medical College, Tianjin, China

**Keywords:** hematopoietic stem cell, hierarchy, heterogeneity, differentiation

## Abstract

Ever since hematopoietic stem cells (HSCs) were first identified half a century ago, their differentiation roadmap has been extensively studied. The classical model of hematopoiesis has long held as a dogma that HSCs reside at the top of a hierarchy in which HSCs possess self-renewal capacity and can progressively give rise to all blood lineage cells. However, over the past several years, with advances in single cell technologies, this developmental scheme has been challenged. In this review, we discuss the evidence supporting heterogeneity within HSC and progenitor populations as well as the hierarchical models revised by novel approaches mainly in mouse system. These evolving views provide further understanding of hematopoiesis and highlight the complexity of hematopoietic differentiation.

## THE CLASSICAL AND BALANCED HEMATOPOIETIC HIERARCHICAL MODEL

The cellular potential of hematopoietic stem cells (HSCs) has been traditionally defined by transplanting donor cells (or a single cell) into recipients that are preconditioned by lethal irradiation and therefore devoid of a functional endogenous hematopoietic system. This assay has long been the gold-standard for functional HSCs.

The first *in vivo* evidence for the existence of HSCs, in 1961, was based on the rescue of lethally irradiated recipient mice by bone marrow transplantation, followed by observing hematopoietic colonies in the spleens of recipients (Till and Mc, 1961). Thereafter, scientists were interested in developing methods to purify HSCs from bone marrow to better understand their function and molecular regulatory networks. Separation of HSCs became possible with the utilization of antibodies and fluorescence-activated cell sorting (FACS). Weissman and colleagues first described HSC-enriched cells using the combination of several surface markers in 1988 (Spangrude et al., 1988). Since then, different groups have put great effort into identifying more surface markers to further purify HSCs. To date, CD34, Sca-1, c-Kit, the signaling lymphocyte activation molecule (SLAM) markers, etc. are still commonly used to isolate HSCs in different labs (Ikuta and Weissman, 1992; Okada et al., 1992; Osawa et al., 1996; Kiel et al., 2005; Oguro et al., 2013). Since similar approaches can be used to identify multi- and unipotent progenitors, different progenitor populations were also isolated based on surface markers (Kondo et al., 1997; Akashi et al., 2000; Adolfsson et al., 2005; Wilson et al., 2008; Pietras et al., 2015).

**Figure 1:**
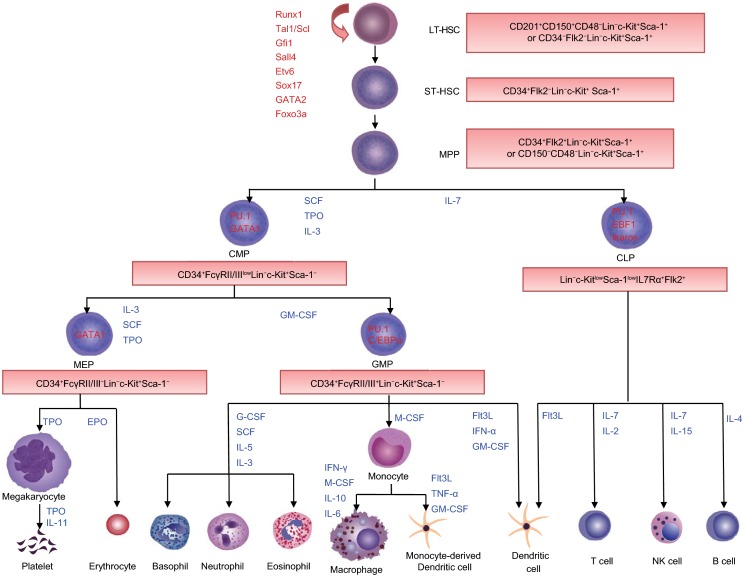
The classical hematopoietic hierarchy. In the classical model, LT-HSCs sit at the top of hierarchy. LT-HSCs differentiate into ST-HSCs, and subsequently to MPPs with reduced self-renewal ability. Downstream of MPPs, a strict separation between the myeloid (CMPs) and lymphoid (CLPs) branches is the first step in lineage commitment. CMPs can generate MEPs and GMPs. CLPs give rise to lymphocytes and dendritic cells. MEPs differentiate into megakaryocytes/platelets and erythrocytes. GMPs produce granulocytes, macrophages, and dendritic cells. Hematopoietic differentiation is controlled by extrinsic cytokines and intrinsic transcription factors

Through transplantation and colony assay, HSCs have been defined on the basis of two essential properties, self-renewal and multipotent differentiation, which can produce cells of all blood lineages (Morrison et al., 1995; Orkin, 2000; Reya et al., 2001; Dick, 2003; Reya, 2003). By contrast, progenitors have been defined by the absence of self-renewal and restricted lineage differentiation capacities. To better illustrate the relationship between an HSC and its progenies, and the stepwise differentiation process, the immunophenotype-based tree-like hierarchy model was largely established by Weissman’s group (Kondo et al., 1997; Morrison et al., 1997; Akashi et al., 2000; Manz et al., 2002). In this classical model, HSCs can be divided into two subpopulations according to their CD34 expression: CD34^−^ long-term (LT)-HSCs and CD34^+^ short-term (ST)-HSCs. LT-HSCs are a rare, quiescent population in bone marrow and have full long-term (> 3~4 months) reconstitution capacity, whereas ST-HSCs only have a short-term (mostly < 1 month) reconstitution ability. LT-HSCs differentiate into ST-HSCs, and subsequently, ST-HSCs differentiate into multipotent progenitors (MPPs), which have no detectable self-renewal ability (Yang et al., 2005). The first bifurcation occurs between the common myeloid progenitors (CMPs, with myeloid, erythroid and megakaryocytic potential) and common lymphoid progenitors (CLPs, with only lymphoid potential), which are derived from MPPs. The second branch point at CMPs segregates bipotent granulocyte-macrophage (GMPs) and megakaryocyte-erythrocyte progenitors (MEPs). CLPs further form T, B, NK and dendritic cells, while GMPs differentiate into granulocytes/monocytes and MEPs generate megakaryocytes/erythrocytes. All these populations form a tree-like and balanced hierarchy model, within which key transcription factors (TFs) and cytokines precisely conduct the stepwise differentiation of HSCs to mature blood cells (Zhu and Emerson, 2002; Robb, 2007; Metcalf, 2008; Zhang and Lodish, 2008; Seita and Weissman, 2010) (Fig. 1).

## ADVANCES IN THE HEMATOPOIETIC HIERARCHY

Although the classical model has been very useful for understanding the differentiation process of HSCs, it is worth noting that this model has some shortcomings in that it oversimplifies the complexity of hematopoietic stem and progenitor cells (HSPCs), and it is only based on the surface markers and transplantation using bulk cells. Bulk cell analysis assumes that each cell, which has the same phenotype, possesses an identical function. With advances in single cell technology and genetic mouse models, this classical model has been challenged over the past several years, especially in the elucidation of megakaryopoiesis. Moreover, new types of HSPCs have been identified and extensively studied due to their lineage biases.

### Heterogeneity in HSCs lineage output and debates on megakaryocyte differentiation

**Figure 2:**
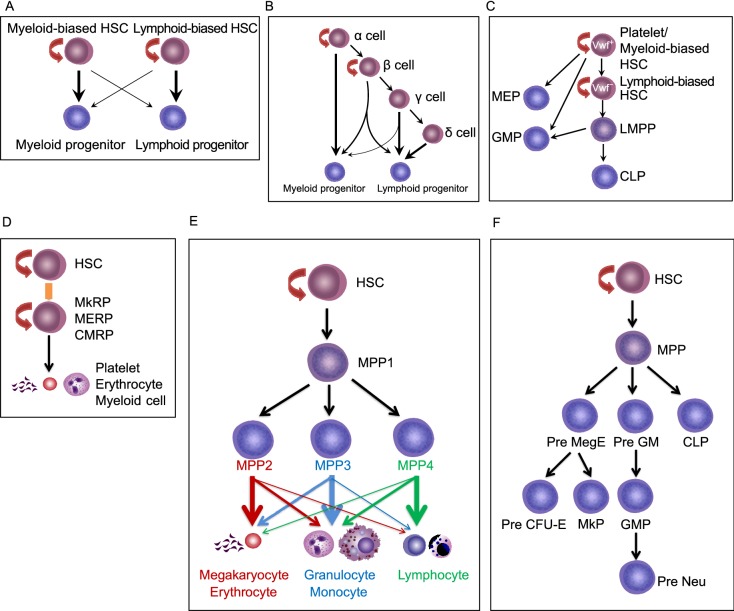
The revised models for hematopoietic stem cell differentiation. (A) My-Bi and Ly-Bi HSCs model. Ly-Bi HSCs reconstitute the myeloid lineage to a lesser extent than the lymphoid lineage, and vice versa. (B) Eaves’ lab defined α, β, γ, and δ cells according to the percentage of myeloid chimerism relative to that of lymphoid chimerism (M/L ratio). Single donor cell is defined as α cells when the M/L ratio exceeds 2, β cells when M/L ratio exceeds 0.25 but is less than 2, and γ/δ cells when it is less than 0.25. Therefore, α cells are myeloid-biased, β cells are balanced, and γ/δ cells are lymphoid-biased without 2nd transplantation capability. (C) vWF^+^ platelet-biased HSCs sit at the apex of the hierarchy, and can differentiate into all progenitors and mature cells. vWF^−^ lymphoid-biased HSCs reside downstream of vWF^+^ HSCs. LMPPs cannot give rise to the megakaryocyte/erythrocyte lineage. MEPs are directly derived from HSCs. (D) In the myeloid bypass model, the LT-HSC population contains CMRPs, MERPs, and MkRPs. These MyRPs are directly produced by HSCs. (E) MPP subtypes are separated into MPP1–4. MPP1 can give rise to all lineages. MPP2/3 are myeloid-biased and MPP4 is lymphoid-biased. In addition, MPP2 is platelet-biased. (F) In this model, MPPs differentiate into pre MegE, Pre GM and CLP. Pre MegE is upstream of MkP and pre CFU-E. Pre GM gives rise to GMP, and subsequently generates newly defined neutrophil precursors (Pre Neu)

By using limiting-dilution analysis and single-cell transplantation, the Sieburg and Eaves groups defined myeloid-biased (My-Bi), balanced (Ba) and lymphoid-biased (Ly-Bi) HSCs based on the ratio of myeloid to lymphoid cells outputs (Muller-Sieburg et al., 2002; Muller-Sieburg et al., 2004; Dykstra et al., 2007; Benz et al., 2012) (Fig. 2A and 2B). In addition, platelet-biased HSCs have also been reported as a My-Bi subset residing at the top of the hematopoietic hierarchy (Sanjuan-Pla et al., 2013) (Fig. 2C). Researchers have long recognized the concept of LT-HSCs and ST-HSCs. Based on the reconstitution time period, intermediate-term HSCs (IT-HSCs), which sit in-between LT-HSC and ST-HSC and contribute to reconstitution up to 8 months after transplantation, have been used in several labs (Benveniste et al., 2010; Yamamoto et al., 2013). In addition, Lu *et al.* tracked single HSCs *in vivo* using viral genetic barcoding combined with high-throughput sequencing (Lu et al., 2011). They also revealed heterogeneity in the HSC population. In this assay, they showed that that HSCs do not equally contribute to progenies, and that two distinct HSC differentiation patterns co-exist in the same recipient mouse after irradiation. One differentiation pattern consists of progenitor cell populations including GMPs, MEPs and CLPs; the other group consists of mature lymphoid blood cells. Similarly, with single cell transplantation, Yamamoto *et al.* observed that self-renewing lineage-restricted progenitors exist in phenotypically defined HSC, containing megakaryocyte repopulating progenitors (MkRPs), megakaryocyte-erythrocyte repopulating progenitors (MERPs), and common myeloid repopulating progenitors (CMRPs) (Yamamoto et al., 2013) (Fig. 2D). This study suggests that oligo-, bi- and unipotent cells co-exist in HSC populations. Furthermore, SLAM family markers CD150 and CD229 can segregate HSCs into different fractions with differentiation reconstitution ability. Compared with CD150^med^ HSC, CD150^hi^ HSC displayed higher self-renewal potential with myeloid biased differentiation (Morita et al., 2010). CD229^−^ HSCs have long-term self-renewal potential with myeloid biased potential and form all of the other stem and progenitor cell populations, whereas CD229^+^ HSCs appear to have less self-renewal capacity with lymphoid biased potential (Oguro et al., 2013). The single-cell omics analyses (Moignard et al., 2013; Wilson et al., 2015; Nestorowa et al., 2016; Buenrostro et al., 2018; Laurenti and Gottgens, 2018; Jacobsen and Nerlov, 2019), including single-cell RNA sequencing (scRNA-seq) and single cell assay for transposase-accessible chromatin using sequencing (scATAC-seq), have further uncovered the presence of heterogeneity in the most primitive HSC populations.

The origin of megakaryocytes has been under debate for several years. The Jacobsen group identified lymphoid-primed MPPs (LMPPs) that give rise to granulocyte/macrophage and lymphoid lineages but not a megakaryocyte/ erythrocyte lineage (Adolfsson et al., 2005) (Fig. 2C). However, lineage tracing studies challenge this view and suggest that LMPPs also differentiate into a megakaryocyte/erythrocyte lineage (Forsberg et al., 2006; Boyer et al., 2011). The different outcomes may be attributed to the cell dose used for transplantation and the methods, including mouse models, used in different labs. More recently, mRNA expression of the megakaryocyte marker von Willebrand factor (vWF) and the surface receptor c-Kit were suggested to be indicative of a platelet-biased, but multipotent HSC sub-population (Sanjuan-Pla et al., 2013; Shin et al., 2014). Evidence for a platelet-biased population of HSCs was provided by the Jacobsen group (Sanjuan-Pla et al., 2013). They found that 25% of LT-HSCs express vWF and vWF^+^ HSCs are primed for platelet-specific gene expression, with enhanced propensity for long-term reconstitution of platelets. The vWF^+^ platelet-primed HSCs also have a long-term myeloid lineage bias, can self-renew, and can give rise to vWF^−^ lymphoid-biased HSCs (Fig. 2C). Therefore, the platelet-primed HSCs sit at the top of the hematopoietic hierarchy. Moreover, based on single-cell transplantation experiments, the existence of megakaryocyte-lineage restricted cells in the phenotypic HSC compartment was proposed (Yamamoto et al., 2013). Pair-daughter cell transplantation assay indicates that megakaryocyte precursors are directly derived from HSCs (Yamamoto et al., 2013) (Fig. 2D). Later, a study reported that the HSC compartment contains stem-like megakaryocyte committed progenitors (SL-MkPs), a cell population that shares many features with HSCs (Haas et al., 2015). This population becomes activated upon inflammatory stress to efficiently replenish platelets, thus a potential shortcut from HSCs to megakaryocytes has been suggested under inflammatory conditions (Haas et al., 2015). Furthermore, by tracking progenitors and mature lineage cells produced from single transplanted HSCs, a recent report from Jacobsen’s lab showed that a distinct class of HSCs adopts a fate towards long-term and effective replenishment of megakaryocytes/platelets without replenishment of any other blood cell lineages, whereas no HSCs contribute exclusively to any other single blood cell lineage (Carrelha et al., 2018).

Collectively, HSCs and megakaryocytes share several features, for example, expression of thrombopoietin receptor (MPL), CD150, CXCR4 and vWF, etc. (Wang et al., 1998; Sugiyama et al., 2006; Pronk et al., 2007; Yoshihara et al., 2007; Huang and Cantor, 2009). More importantly, megakaryocytes also serve as an HSC niche component and tightly regulate the maintenance of HSC function (Bruns et al., 2014; Zhao et al., 2014). Platelet- and myeloid-biased vWF^+^ HSCs, but not lymphoid-biased vWF^−^ HSCs, associate with megakaryocytes and are regulated by megakaryocytes (Pinho et al., 2018). All the evidence suggests that HSCs and the megakaryocyte (or its progenitors) are closer to one another in the hematopoietic developmental hierarchy than previously appreciated. In consideration of the heterogeneity observed in the HSC population, our view is that lineage (cell fate) predetermination occurs in HSCs, prior to their differentiation towards progenitors. Moreover, the megakaryocyte can arise independent of other lineages, and the megakaryocyte differentiation route is first separated from other blood cell lineages in the hierarchy.

### Heterogeneity in MPPs

The Trumpp (Wilson et al., 2008) and Passegue (Pietras et al., 2015) groups further divided the MPP population into MPP1, MPP2, MPP3 and MPP4 according to their immuno-phenotype, cell cycle status, lineage bias, resistance to drug treatment and bone marrow abundance. MPP1 is more similar to the previously defined IT-HSC or ST-HSC, which have multiple-lineage reconstitution ability up to 4 months in the first transplantation, whereas MPP2/3/4 are devoid of self-renewal potential and only exhibit short-term myeloid reconstitution ability (<1 month). More importantly, MPP2 and MPP3 produce low levels of T and B cells and MPP4 generates low levels of myeloid cells *in vivo*. In addition, compared with MPP3 and MPP4, MPP2 produces higher levels of platelets. Taken together, MPP2 is a megakaryocyte-biased MPP subset and MPP3 is a myeloid-biased MPP subset. Both MPP2 and MPP3 are functionally distinct from the lymphoid-primed MPP4. HSCs independently generate all three types of lineage-biased MPPs (MPP2-4), but among them, no MPPs are able to generate other MPPs *in vivo* (Fig. 2E). After transplantation, HSCs first produce myeloid-biased MPPs (MPP1/2) to quickly establish myeloid output, followed by the lymphoid-primed MPP4 subpopulation to rebuild the lymphoid compartment. Therefore, MPPs are a heterogeneous population with different lineage-biased potential both at the cellular and molecular levels.

### Heterogeneity and hierarchy within myeloid progenitors

In the classical model, CMPs and MEPs are separated according to CD34 expression. In the Lin^-^cKit^+^Sca1^-^ (LKS^-^) population, CMPs are CD34^+^CD16/32^-^, whereas MEPs are CD34^-^CD16/32^-^. CMPs are thought to possess oligo-potency, including granulocyte, macrophage, megakaryocyte and erythrocyte differentiation potential. However, CMPs have a low clonal frequency of mixed myeloid colonies, and MEPs also possess a low level of megakaryocyte potential (Nakorn et al., 2003). Therefore, it prompts us to know whether each CMP or MEP has a different lineage potential at the single cell level, i.e., whether every CMP is indeed oligo-potent, and whether MEP is bipotent.

To understand the heterogeneity and lineage commitment in the LKS^-^ myeloid progenitor population, especially in CMPs, Pronk et al. (Pronk et al., 2007) used CD150, CD105 (Endoglin), CD41 and CD16/32 to re-segregate the LKS^-^ myeloid progenitors. In the LKS^-^ population, CD41^+^CD150^+^ cells are defined as megakaryocyte progenitors (MkPs), which are exclusively associated with megakaryocyte generation. CD41^-^CD150^-^ CD16/32^+^ cells are GMPs. In the CD41^-^CD150^-^CD16/32^-^ population (classical CMPs and MEPs mixture), there are four newly defined sub-populations, including pre MegEs, pre GMs, Pre CFU-Es and CFU-Es (Pronk et al., 2007). Single Pre MegE cells can effectively produce megakaryocytic, erythroid as well as mixed megakaryocyte/erythroid colonies. In contrast, Pre CFU-E cells give rise almost exclusively to erythroid colonies of various sizes. Pre GMs lie developmentally upstream of GMPs, and have a remarkably similar clonal lineage output to the GMPs. Therefore, the Pronk *et al.* study explores the processes of myeloid cell differentiation, reveals a number of novel intermediate progenitors, and orchestrates a new hierarchy model, including unipotent proliferative neutrophil precursors (Kim et al., 2017; Evrard et al., 2018; Zhu et al., 2018). Classical CMPs consist of pre GMs, and the majority of pre MegEs and MEPs are separated into CFU-E, Pre CFU-Es, and part of pre MegEs. Moreover, MkPs are located mainly in CMPs (Pronk et al., 2007) (Fig. 2F).

In line with the work discussed above, a landmark paper from Amit’s lab reporting the transcriptomes for more than 2,600 mouse single LKS^-^ myelo-erythroid progenitor cells (Paul et al., 2015) and a subsequent work from Gottgens’ lab reporting the transcriptomes of 1,600 HSPCs (Nestorowa et al., 2016) both revealed heterogeneity in LKS^-^ progenitors. The single cells from classical MEPs do not show any expression of megakaryocyte markers or prominent megakaryocyte TFs. However, both megakaryocyte markers (Pf4 and CD41) and TFs (Pbx1, Fli1, Mef2c) are expressed in the cells from classical CMPs (Paul et al., 2015).

Taken together, all the studies explained why megakaryocytes mainly differentiate from CMPs, but not MEPs, and MEPs mostly give rise to erythrocytes. Therefore, this suggests that classical MEPs may not be the true precursor for megakaryocytes.

### Hematopoietic differentiation is a continuous process

**Figure 3:**
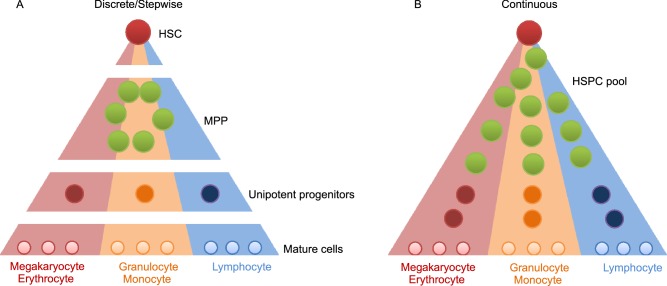
Discrete vs. continuous hematopoietic differentiation model. (A) The discrete differentiation model shows that HSCs differentiate to mature lineage the progression cells is a stepwise process following a tree-like hierarchy of oligo-, bi- and unipotent progenitors. (B) The continuous differentiation model shows that there is no obvious boundary in the hierarchy. Individual HSCs gradually acquire lineage biases along multiple directions without passing through discrete hierarchically organized progenitor populations

Previous studies indicated that individual HSCs gradually acquire lineage biases along multiple directions while passing through discrete hierarchically organized progenitor populations (Fig. 3A). However, these models are based on the analysis of predefined flow-sorted cell populations. With advances in methodologies, it has become possible to study the similarities or differences of individual HSPCs and their differentiation relationships.

By using scRNA-seq combined with computational analysis, a recent study on comprehensively sampled human bone marrow HSPCs suggested a model in which acquisition of lineage-specific fates is a continuous process, and unilineage restricted cells emerge directly from a continuum of low-primed undifferentiated HSPCs, without any major transition through the multi- and bi-potent stages (Velten et al., 2017). This view is supported by a zebrafish study that suggested that the continuum of HSPC differentiation is characterized by a highly coordinated transcriptional program, displaying simultaneous suppression of cell proliferation-related genes and upregulation of lineage specific genes (Macaulay et al., 2016). In addition, another study using human cord blood lympho-myeloid progenitor cells, including LMPPs, GMPs and multi-lymphoid progenitors (MLPs), further suggested a model in which a continuum of progenitors execute lymphoid and myeloid differentiation, rather than only unilineage progenitors being present downstream of stem cells (Karamitros et al., 2018). Although most progenitors have uni-lineage potential, bi- and oligo-lineage progenitors are present among LMPPs, GMPs and MLPs. These aforementioned studies change our view that hematopoietic differentiation is a continuous process, rather than a discrete hierarchy, which suggests that there is no obvious boundary among stem cells and progenitors (Laurenti and Gottgens, 2018) (Fig. 3B).

### The roadmap of human hematopoiesis

In human hematopoiesis, the Dick group sorted MPPs, CMPs and MEPs from fetal liver and adult bone marrow, and compared their lineage potential from different developmental stages (Notta et al., 2016). They showed that previously defined MPPs, CMPs and MEPs are heterogeneous. Importantly, MEPs, from both fetal liver and bone marrow, uniformly produce erythroid-only clones. Therefore, the classically defined MEPs are principally erythroid precursors when analyzed at single cell resolution and are not megakaryocyte/erythroid progenitors as previously thought, which is consistent with the observation in a mouse model (Pronk et al., 2007). Interestingly, fetal liver contains large numbers of distinct oligopotent progenitors. However, few oligopotent progenitors were present in the adult bone marrow. Instead only two progenitor classes predominate, multipotent and unipotent, with megakaryocyte/erythroid lineages emerging from multipotent cells. The Dick group’s study provides a revised model to understand normal hematopoiesis, which is indeed flexible in developmental time.

## HEMATOPOIESIS UNDER PHYSIOLOGICAL CONDITION

Hematopoiesis is regulated by microenvironment or niche (Morrison and Scadden, 2014; Crane et al., 2017), therefore, an unperturbed niche and a pretreated niche have different effects on hematopoiesis (Mendelson and Frenette, 2014). LT-HSC is considered to sit at the apex of the hierarchy, and reconstitutes or maintains the whole hematopoiesis. However, it is important to remember that almost all the supporting evidence was obtained from *in vitro* colony assay and *in vivo* transplantation. Irradiation or drug treatment can disrupt the niche, ablate the hematopoietic cells in the recipient, and create the space for donor HSPC engraftment, expansion and differentiation. Moreover, HSPCs are retained in the niche under hypoxic regulation (Suda et al., 2011; Nombela-Arrieta et al., 2013; Spencer et al., 2014; Itkin et al., 2016), and it is reported that transient exposure of HSPCs to normal oxygen impairs their function (Mantel et al., 2015). Therefore, transplantation of HSPCs into pretreated recipients cannot truly reflect the behaviors of hematopoiesis under physiological conditions (steady state).

To understand the dynamics of blood formation in steady state, various lineage-tracing approaches have been used to assess the lineage contribution of individual HSPCs in unperturbed hematopoiesis. By using a doxycycline-induced Sleeping Beauty transposon tagging approach in HSPCs, Sun *et al.* (Sun et al., 2014) reported that MPPs, rather than HSCs, are the main drivers of steady-state hematopoiesis during adulthood. In addition, Rodewald’s lab devised a mouse model allowing inducible genetic labeling of the most primitive Tie2^+^ HSCs in bone marrow, and quantified label progression along hematopoietic development by limiting dilution analysis and mathematical modelling (Busch et al., 2015). They found that adult hematopoiesis is largely sustained by ST-HSC, and in contrast, LT-HSCs are rapidly used to establish the immune and blood system in fetal and early postnatal life. Another study from the same group used an alternative genetic fate-mapping system, called polylox barcoding (Pei et al., 2017), and demonstrated that when HSCs are labeled at the fetal liver stage, their descendants in the adult will mostly contribute to multiple lineages. However, when the analysis was repeated in adult stage, few barcodes were detected in HSCs as well as mature progenies. The above studies support the view that MPPs or ST-HSCs contribute predominantly to mature progenies, whereas HSCs do not have a notable role in steady-state hematopoiesis. However, Sawai et al. (2016) reported a paradoxical result. Pdzk1ip1 (Map17) is specifically expressed in the murine HSC population, therefore, they developed Pdzk1ip1-GFP and Pdzk1ip1-CreER; R26-TdTomato mice and showed that LT-HSCs provide a major contribution to all lineage-committed progenitors and mature blood cells. These controversial observations might be owing to the differences of tracing methods and labeling efficiency in HSCs. Therefore, new approaches should be developed and further investigation will be necessary to reconcile this paradox.

Interestingly, in Rodewald’s polylox tracking study, they revealed a basic split between common myeloid–erythroid development and common lymphocyte development, supporting the bifurcating tree model of hematopoiesis that has not been proved in steady state (Pei et al., 2017). However, megakaryocytic fate was not analyzed in this study. Another study from the Camargo group addressed this issue more comprehensively (Rodriguez-Fraticelli et al., 2018). They performed a long-term (30-week) pulse-chase experiment in adult mice with the sleeping beauty barcode system, and found that during unperturbed hematopoiesis, the megakaryocyte lineage arises largely independently of other hematopoietic fates, and the LT-HSCs predominantly contribute to megakaryocyte output.

## CONCLUDING REMARKS

**Figure 4:**
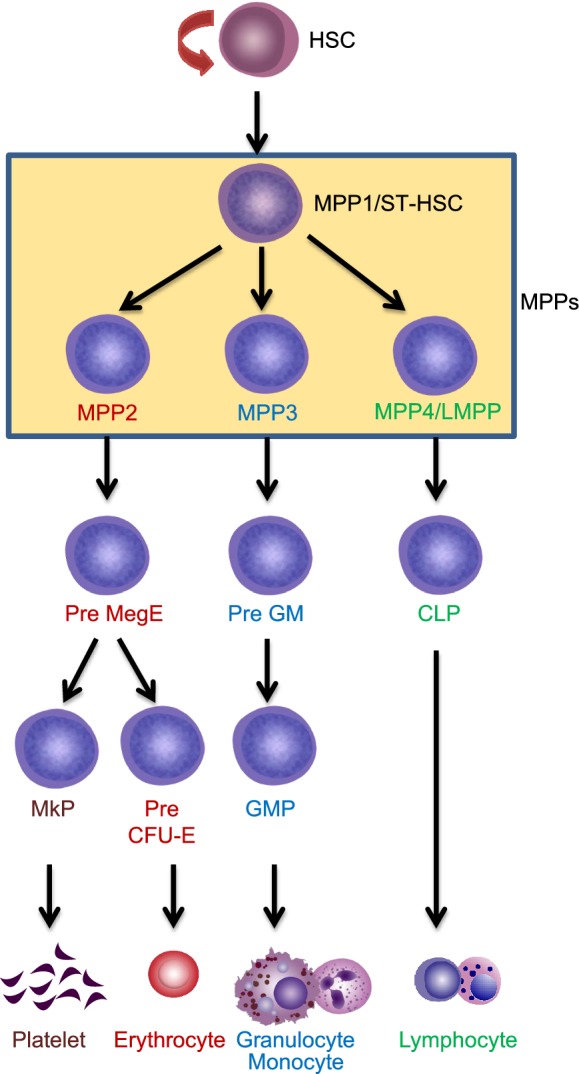
A reconciled model for hematopoietic stem cell differentiation. In this model, HSCs first differentiate into MPP1/ST-HSC, then give rise to MPP2, MPP3 and MPP4 (LMPP). MPP2 can generate pre MegE, and subsequently, pre MegE gives rise to platelets through MkP or produce erythrocytes through Pre CFU-E. MPP3 mostly give rise to granulocyte and monocyte lineages, and MPP4 (LMPP) mainly contribute to lymphocytes

Advanced technologies and new findings have broadened our knowledge on hematopoiesis. Hematopoietic hierarchy is more complicated than what we previously thought. In consideration of all the findings discussed above, the model shown in Fig. 4 represents the ideal hierarchy so far. However, we believe that with the advances in single cell technology, more subtypes of stem and progenitor cells will be discovered. Moreover, the epigenetic status of single HSPCs analyzed by single cell Hi-C (Nagano et al., 2013) and single cell ATAC-seq (Buenrostro et al., 2018; Cao et al., 2018; Cusanovich et al., 2018; Satpathy et al., 2018) will provide more information and the hierarchical model will be further revised. By combining imaging with *in situ* RNA-seq (Wang et al., 2018; Eng et al., 2019; Rodriques et al., 2019), studying the spatial localization of individual HSCs or HPCs becomes possible. This can help us know whether single HSPCs, with different transcriptomes, have specific lodgment sites in bone marrow.

The kinetics of adult HSC differentiation under different stress conditions have been studied recently in great detail (Lu et al., 2019). However, whether expansion of HSCs in recipients after transplantation occurs is still poorly understood. Furthermore, future studies can focus more on the changes of heterogeneity and hierarchy of HSPCs in disease. For example, one study reported that a c-Kit^hi^ progenitor subset positive for IL-7Rα emerged after infection of mice with *Plasmodium chabaudi* (Belyaev et al., 2010). These cells have both lymphoid and myeloid potential. Therefore, studying HSPCs under different diseases is interesting and also important.

## Abbreviations

Ba: balanced
CLPs: common lymphoid progenitors
CMPs: common myeloid progenitors
CMRPs: common myeloid repopulating progenitors
FACS: fluorescence-activated cell sorting
GMPs: granulocyte-macrophage progenitors
HSCs: hematopoietic stem cells
HSPCs: hematopoietic stem and progenitor cells
IT-HSCs: intermediate-term hematopoietic stem cells
LKS^-^: Lin^-^cKit^+^Sca1^-^
LMPPs: lymphoid-primed multipotent progenitors
LT-HSCs: long-term hematopoietic stem cells
Ly-Bi: lymphoid-biased
MEPs: megakaryocyte-erythrocyte progenitors
MERPs: megakaryocyte-erythrocyte repopulating progenitors
MkPs: megakaryocyte progenitors
MkRPs: megakaryocyte repopulating progenitors
MLPs: multi-lymphoid progenitors
MPPs: multipotent progenitors
My-Bi: myeloid-biased
scATAC-seq: single cell assay for transposase-accessible chromatin using sequencing
scRNA-seq: single-cell RNA sequencing
SLAM: signaling lymphocyte activation molecule
SL-MkPs: stem-like megakaryocyte committed progenitors
ST-HSC: short-term hematopoietic stem cells
TFs: transcription factors
vWF: von Willebrand factor
